# Cancer Immunotherapy and Delivery System: An Update

**DOI:** 10.3390/pharmaceutics14081630

**Published:** 2022-08-04

**Authors:** Ming Yang, Olamide Tosin Olaoba, Chunye Zhang, Eric T. Kimchi, Kevin F. Staveley-O’Carroll, Guangfu Li

**Affiliations:** 1 Department of Surgery, University of Missouri, Columbia, MO 65212, USA; 2 Harry S. Truman Memorial VA Hospital, Columbia, MO 65201, USA; 3 Department of Molecular Microbiology and Immunology, University of Missouri-Columbia, Columbia, MO 65212, USA; 4 Department of Veterinary Pathobiology, University of Missouri, Columbia, MO 65212, USA; 5 Ellis Fischel Cancer Center, University of Missouri, Columbia, MO 65212, USA

**Keywords:** cancer immunotherapy, delivery systems, nanoparticles, biomaterials, T cell therapy, intratumoral delivery, clinical application

## Abstract

With an understanding of immunity in the tumor microenvironment, immunotherapy turns out to be a powerful tool in the clinic to treat many cancers. The strategies applied in cancer immunotherapy mainly include blockade of immune checkpoints, adoptive transfer of engineered cells, such as T cells, natural killer cells, and macrophages, cytokine therapy, cancer vaccines, and oncolytic virotherapy. Many factors, such as product price, off-target side effects, immunosuppressive tumor microenvironment, and cancer cell heterogeneity, affect the treatment efficacy of immunotherapies against cancers. In addition, some treatments, such as chimeric antigen receptor (CAR) T cell therapy, are more effective in treating patients with lymphoma, leukemia, and multiple myeloma rather than solid tumors. To improve the efficacy of targeted immunotherapy and reduce off-target effects, delivery systems for immunotherapies have been developed in past decades using tools such as nanoparticles, hydrogel matrix, and implantable scaffolds. This review first summarizes the currently common immunotherapies and their limitations. It then synopsizes the relative delivery systems that can be applied to improve treatment efficacy and minimize side effects. The challenges, frontiers, and prospects for applying these delivery systems in cancer immunotherapy are also discussed. Finally, the application of these approaches in clinical trials is reviewed.

## 1. Introduction

Commonly, cancer therapeutics include surgery, chemotherapy, hormone therapy, hyperthermia, immunotherapy, photodynamic therapy, radiation therapy, stem cell transplant, and targeted therapy. Cancer immunotherapy is a group of therapeutic strategies that harness immunity to combat cancer growth and metastasis. The strategies applied in cancer immunotherapy mainly consist of blockade of immune checkpoints, adoptive transfer of engineered cells (T cells, natural killer/NK cells, and macrophages), cytokine therapy, cancer vaccines, and infection of oncolytic viruses [1]. Clinical evidence shows that cancer immunotherapy has become an effective therapy against many cancers, such as triple-negative breast cancer (TNBC) [2,3], hepatocellular carcinoma (HCC) [4,5,6], and melanoma [7,8]. It can be used as a first-line treatment for patients with metastatic or surgically unresectable tumors [9]. For example, in 2015, pembrolizumab was approved by the U.S. Food and Drug Administration (FDA) for the treatment of patients with metastatic non-small cell lung cancer (NSCLC) expressing programmed death-ligand 1 (PD-L1) on disease progression or after platinum-containing chemotherapy or targeted therapy [10]. In 2016, pembrolizumab was also approved by the FDA for the treatment of patients with recurrent or metastatic head and neck squamous cell carcinoma (HNSCC) on disease progression or after platinum-containing chemotherapy [11].

Despite the fast-increasing development of cancer immunotherapy, only limited clinical benefits have been shown in the treatment of some cancers. For example, a randomized, multi-center phase 3 clinical study (CheckMate 459), after a minimum follow-up of 33.6 months, showed that the overall survival (OS) rates of patients with advanced HCC (aHCC) after treatment with nivolumab and sorafenib were 29% (95% confidence interval/CI, 25–34) and 21% (95% CI, 17–25), respectively [12]. In addition, immune-related adverse events have been observed in patients with cancer immunotherapy, such as the treatment of immune checkpoint blockers [13]. For example, immune-mediated acute hepatitis was observed in 16 of 536 patients (2.985%) with metastatic cancer. Among these 16 patients, eight were treated with anti-programmed cell death protein 1 (PD-1) monoclonal antibodies (mAbs) and one with anti-PD-L1 mAbs, and seven patients received anti-cytotoxic T lymphocyte antigen 4 (CTLA-4) mAbs [14]. In addition, factors such as immunosuppressive tumor microenvironment (TME) induced by infiltration of immunosuppressive cells and/or upregulated expression of immune checkpoints, cancer cell heterogeneity, and lack of antigen presentation can suppress the efficacy of cancer immunotherapy [15,16]. Therefore, improving treatment efficacy and reducing the side effects of cancer immunotherapy are critically important.

Drug or cell delivery systems have been broadly applied to increase the efficacy of immunotherapy and reduce untargeted cytotoxicity by working as an integrated platform to deliver individual therapies or multiple treatments and to modulate different immune responses against cancer cells [17]. Commonly used delivery systems include nanoparticles (NPs), cell-based delivery, antigen-delivery system, extracellular vesicles-based delivery, hydrogel, and therapeutic cargos and scaffolds that have been broadly applied to the targeted delivery of drugs. For example, dendritic cells (DCs), antigen-presenting cells (APCs), can acquire, process, and present tumor-specific antigens to T cells to induce an anti-tumor immune response [18]. An NP-based mRNA vaccine, encoding tumor antigens to DCs, is able to stimulate an antigen-specific, cytotoxic T lymphocyte response against TNBC in vivo [19].

This review first summarizes the currently common immunotherapies and their limitations. It then synopsizes the relative delivery systems that can be applied to improve treatment efficacy and minimize side effects. Finally, the application of these approaches in clinical trials is reviewed.

## 2. Cancer Immunotherapy

**Figure 1:**
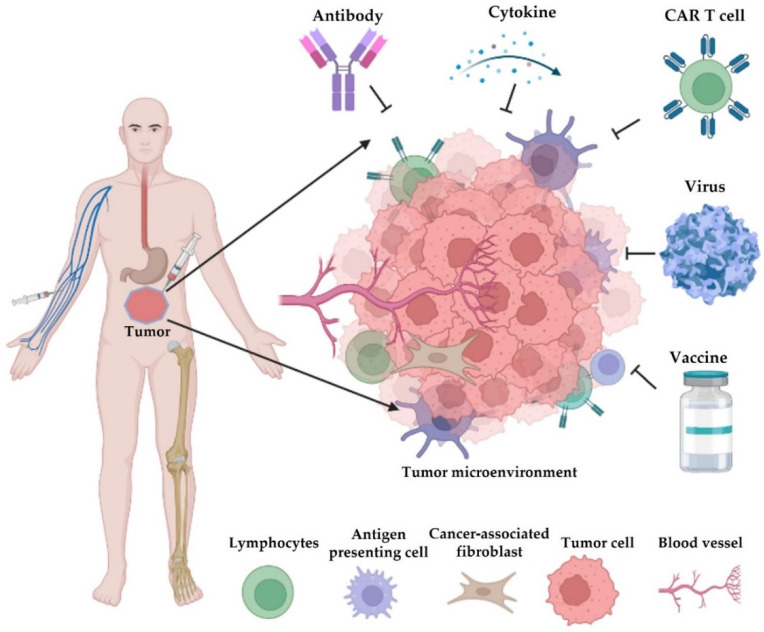
Different types of cancer immunotherapy. They mainly consist of blockade of immune checkpoints (e.g., antibody), adoptive transfer of engineered cells (e.g., chimeric antigen receptor (CAR) T cells, natural killer/NK cells, and macrophages), cytokine therapy, infection of oncolytic viruses, and cancer vaccines. Most of these therapeutics are administered by intravenous injection (i.v.), and some drugs are given by subcutaneous (s.c.), intraperitoneal (i.p.), or intramuscular (i.m.) injections.

Nearly a decade after Science named ’cancer immunotherapy’ as the breakthrough of the year 2013 [20], it has seen remarkable advances over the years. Many preclinical studies yielded novel therapies that became successful upon enrollment in clinical trials. Such success is striking in solid tumors [21]. Thus, immunotherapy is a powerful clinical strategy for the treatment of various diseases, including cancer [22], and an understanding of cancer immunology is important to the optimization of this strategy to achieve higher efficacy. For instance, advancements in single-cell RNA sequencing technologies have provided the opportunity to dissect heterogeneous tumor cells. Interrogation of this TME milieu has given clues to the precise nature of tumor-infiltrating cells and other intratumoral immune cells. Moreover, cancer immunotherapy can manipulate the immune system to identify and fight cancer cells, thereby inducing a durable response [23] with the overall aim of providing active or passive immunity against tumors [24]. Over the years, oncologists have depended on only three treatment options: surgical resection, radiotherapy, and chemotherapy. In addition, the use of small molecule inhibitors for certain kinases in many clinical procedures is still an ongoing practice in precision oncology. However, the emergence of immune-based cancer therapy has improved the choice of treatment and cancer management strategies. Since the discovery and use of the first immunotherapy, scientists have developed several other immunotherapies, including immune checkpoint inhibitors (ICIs), adoptive cell transfer, cytokines, vaccines, and others (Figure 1), which are discussed in detail in this section of the review.

### 2.1. Checkpoint Inhibitors

The discovery of immune checkpoints, such as CTLA-4 and PD-1, has revolutionized cancer immunotherapy [25]. These checkpoints interact with their cognate ligands on tumors and quell antitumor T cell responses. A paradigm for T cell activation includes an initial presentation of a major histocompatibility complex (MHC)-anchored antigen to T cells, interaction with the co-stimulatory receptor, and cytokine stimulation. Another inhibitory co-receptor exists to provide a negative regulation of T cell activation. These inhibitory co-receptors are checkpoint proteins that can induce adaptive tolerance and T cell exhaustion [26]. Notably, immune checkpoints regulate the tumor-killing effect of immune effector cells. Thus, ICIs can target the dysfunctional immune system to restore the effector function of cytotoxic CD8 T cells [27,28].

The first identified immune checkpoint was CTLA-4 [29]. This receptor usually out-competes another cell surface receptor, CD28, on T cells for their costimulatory ligands, CD80 and CD86. Antibodies targeted against CTLA-4 can enhance T cell response to tumors. Ipilimumab is a monoclonal antibody and the first globally approved anti-CTLA-4 for the first or second line of treatment in patients with malignant melanoma [30]. Apart from the use of ipilimumab as a single-agent monotherapy, it has been used in combination with other therapies for the treatment of various malignancies [31,32,33]. Many combination strategies that can block CTLA-4 or other immune checkpoints have been evaluated in several clinical trials around the globe. Mechanistically, anti-CTLA-4 induces preferential ligation of CD80/CD86 to CD28, leading to T cell activation.

PD-1 is another key checkpoint receptor that can modulate T cell activities in order to promote self-tolerance and activate the senescence of antigen-dependent T cells while preventing the apoptosis of regulatory T cells (Tregs). Cancer cells incessantly explore this mechanism by upregulating PD-L1, a cognate ligand of PD-1. Immunotherapy based on PD-1 blockade has shown promising efficacy in both solid and hematological malignancies [34]. In 2014, the FDA approved anti-PD-1 nivolumab, a fully humanized immunoglobulin G4 monoclonal antibody, for the treatment of advanced melanoma [34,35]. This immunotherapy can transform patient cohorts with microsatellite unstable colorectal cancer [36]. Since its initial approval, nivolumab has been repurposed and approved for the treatment of other malignancies. These include NSCLC, renal cell cancer, Hodgkin’s lymphoma, squamous head and neck cancer, urothelial carcinoma, and HCC [37,38]. More recently, the FDA approved cemiplimab (PD-1 inhibitor) for the first-line treatment of advanced non-small cell lung cancer [39], which has been approved for patients with metastatic cutaneous squamous cell carcinoma (CSCC) or patients with locally advanced CSCC that is not suitable for curative surgery or radiation [40].

Another humanized monoclonal anti-PD1 antibody is pembrolizumab. Following its previous approval for the treatment of NSCLC and unresectable melanoma [41], the U.S. FDA approved this ICI for the treatment of patients with advanced PD-L1-positive gastric and gastroesophageal junction adenocarcinoma who have progressed on at least two lines of chemotherapies [42]. A recent report has shown that pembrolizumab can be used as the first line of treatment for recurrent and metastatic HNSCC [43]. In a clinical trial, pembrolizumab showed safety and efficacy signals in phases 1 and 2 for the treatment of classic Hodgkin’s lymphoma [44].

Immunotherapies that can target the PD-1 ligand, PD-L1, have been developed. Atezolizumab is anti-PD-L1 immunotherapy, and the first ICI approved for the treatment of triple-negative breast cancer [45]. Current preclinical and clinical evidence suggests that atezolizumab may be approved as a single monotherapy or in combination with other therapies for the treatment of various malignancies. In addition to atezolizumab, two additional anti-PD-L1 immunotherapies have been approved. One, durvalumab, was approved for the treatment of urothelial cancer [46] and extensive-stage SCLC patients [47]. The other, namely avelumab, is a human IgG1 approved for the treatment of Merkell cell carcinoma and urothelial carcinoma [48].

### 2.2. Cytokine Therapies

Cytokines play an important role in the regulation of innate and adaptive immunity while acting as messengers via autocrine and paracrine signalings over a short distance [49]. Certain antitumor effector functions involving critical aspects of immunity require the release of cytokines or cytokine-mediated activation of antitumor immunity. Over time, scientists have developed an interest in harnessing cytokines for the treatment of cancer.

Interferon alpha (IFN-α) belongs to the family of cytokines. Like other type I IFNs, it signals through the Janus kinase 1 (JAK1) signal transducer and activator of the transcription (STAT) pathway. IFNα polarizes CD4 T cells to T helper type 1 (Th1) effector cells, upregulates MHC class I molecules, and activates caspase-dependent apoptosis in certain cancers. For decades, various formulations of recombinant IFN-α were approved for the treatment of various malignancies, including metastatic renal cell carcinoma, acquired immunodeficiency syndrome (AIDs)-related Kaposi’s sarcoma, follicular lymphoma, chronic myelogenous leukemia, cervical intraperitoneal neoplasms, and completely resected stage III or IV high-risk melanoma [49].

Another universally approved cytokine therapy is interleukin (IL)-2, which is mainly secreted by Th1 effector cells. CD8 T cells and NK cells also secrete IL-2 but to a lesser extent [50]. While acting as a T cell growth factor, IL-2 promotes the expansion of T cells, which is important in the regulation of T cell response and the maintenance of self-tolerance via activation-induced cell death (AICD). IL-2 has been approved for the treatment of metastatic melanoma and metastatic renal cell carcinoma (mRCC). In addition, IL-2 is widely combined with adoptive T cell therapy, as it enhances the ex vivo expansion of T cells [51,52].

Other cytokines, including IFN-γ, granulocyte-macrophage colony-stimulating factor (GM-CSF), IL-12, IL-15, and IL-21, have been evaluated for their anticancer potential in preclinical and clinical models. IFN-γ showed an initial promising result in a phase 2 trial but was not approved to treat cancer patients due to the lack of efficacy [53]. In several trials, GM-CSF demonstrated inconsistent efficacy in addition to its scarring effect [54]. With a previous promising phase 1 trial result, IL-12 belied the previous dosing regimen and showed adverse effects and mortality [55]. Further, the antitumor efficacy of IL-15 was evaluated in preclinical studies and phase 1/2 clinical trials. IL-15 activated and caused the expansion of NK, NKT, and (m) CD8 T cells [56]. In another study, the combination of IL-15 with anti-PD-L1 and anti-CTLA4 contributed to favorable OS [57]. Thus, the outcomes from these studies are promising for the future development of IL-15–based therapy. On the other hand, IL-21 plays a critical role in chronic inflammatory bowel disease (IBD) and inflammation-induced colon cancer, thus leading to its termination in the clinical trial [58]. Despite these treatment-related adverse events (TRAEs), there are several ongoing clinical trials to re-evaluate the anti-tumor potentials of these cytokines.

### 2.3. Adoptive Cell Transfer Therapy

Adoptive cell therapy (ACT) has emerged as an important therapy for cancers, especially personalized cancer therapy [59]. T cells for ACT mainly include tumor-infiltrating lymphocyte-derived T cells or genetically engineered T cells with the expression of conventional T cell receptors or chimeric antigen receptors [60]. Commonly used gene-editing strategies in T cells include retroviral or lentiviral transduction, zinc finger, or transcription activator-like effector nucleases, and clustered regularly interspaced short palindromic repeat (CRISPR)-associated 9 (Cas9) endonuclease technology [61,62,63].

Chimeric antigen receptor T (CAR-T) cells use a gene transfer technology that involves an ex vivo modification of T cells and an adoptive transfer of the engineered T cells in order to target tumor-associated antigen (TAA) and bolster the antitumor function of T lymphocytes. Although different types of T cells may present different efficacy with a specific CAR technology, various modifications are available to prolong survival and redirect the specificity and function of T cells [64].

In general, CAR-T cells are structurally engineered to contain an extracellular antigen-binding domain derived from a single-chain variable fragment (ScFv) of a monoclonal antibody, an extracellular region containing a spacer domain, a transmembrane domain, and an intracellular domain [65]. Upon antigen binding to the ScFv, the intracellular domain is capable of initiating signaling that culminates in T cell activation. This T cell activation is MHC-independent and can lead to tumor destruction. The success of CAR-T-based immunotherapy depends on the TAA selected for CAR specificity. Usually, the CAR gene is designed to recognize only TAAs that are critical to the survival of the tumor. Despite the promising outcomes of CAR technology, certain tumor genetic mutations and epigenetic alterations are drivers of immunoediting, which can result in therapeutic resistance [66,67].

CAR-T cell-based therapy has shown encouraging efficacies toward hematological malignancies. One major TAA target is CD-19. Kymriah and Yescarta are CD19-targeting CAR-T cell products approved by the U.S. FDA for the standard of care of B cell acute lymphoblastic leukemia (B-ALL) and diffuse large B-cell lymphoma (DLBCL), respectively [68,69]. Brexucabtagene autoleucel (brexu-cel) was also approved for the treatment of relapsed or refractory (r/r) mantle cell lymphoma [70]. Other CD-19-directed CAR-T cell products include tisagenlecleucel (tisa-cel) and axicabtagene ciloleucel (axi-cel), which were approved for the treatment of patients with r/r DLBCL, B-ALL, and primary mediastinal B-cell lymphoma (PMBCL) [71,72]. In other tumor types, CAR may be engineered to recognize other antigens. For instance, ganglioside GD2 is a TAA in neuroblastoma [73]; CD70 is a novel target in gliomas [74]; CD20 and CD22 are TAAs in relapsed refractory Burkitt lymphoma [75]. In our previous study, we reported liver-intestine cadherin (CDH17) as a novel target in pancreatic cancer. Our data showed that knockout of CDH17 suppressed Panc02-H7 growth and caused tumor regression in our orthotopic mouse model [76]. As the evolution of CAR technology continues, CDH17 has become a novel TAA for the development of newly engineered CAR T cells. Feng et al., in a 2022 report, demonstrated that CDH17CAR T cells suppressed neuroendocrine and gastrointestinal tumors without TRAEs [77].

### 2.4. Oncolytic Virotherapy

The use of oncolytic viruses in the treatment of malignancies is becoming increasingly promising. Oncolytic viruses are genetically modified viruses that can selectively replicate and target cancer cells for destruction without any deleterious effects on normal tissue [78]. Mechanistically, virotherapy induces specific antitumor immunity in the context of tumor-specific viral replication.

The first oncolytic virotherapy, rigvir, was developed from the native ECHO-7 strain of picornavirus. Rigvir was registered in Latvia, Georgia, Armenia, and Uzbekistan, where it was approved for the treatment of melanoma [79]. Furthermore, a genetically engineered adenovirus, oncorine (H101), was approved in November 2005 by the Chinese SFDA as a standard of care for nasopharyngeal carcinoma, but in combination with chemotherapy [80]. Other oncolytic adenovirus therapies that can target and eliminate cancer cells in both preclinical and clinical models have been developed [81,82,83]. However, the efficacy of this therapy is generally challenged by inefficient systemic delivery to the target tissue. This is due to the presence of pre-existing neutralizing antibodies to adenovirus [84] and non-specific uptake [85]. In 2015, a herpes simplex virus type 1 (HSV-1)-based virotherapy was developed. This modified HSV-1, called Talimogene laherparepvec (T-vec), was armed with GM-CSF. Following a phase 3 clinical trial that showed that T-vec significantly caused tumor regression and prolonged the OS of melanoma patients [86], the U.S. FDA approved T-vec for the treatment of unresectable cutaneous, subcutaneous, and nodal lesions in melanoma patients with relapse [78,87].

In addition to the approved virotherapies, at least 40 oncolytic viruses are currently being tested against different cancers [88]. Some of these oncolytic viral therapies have shown promising results in phase 3 clinical trials and are now awaiting approval. These include oncolytic vaccinia virus-derived pexastimogen devbacirepvec (pexa-vec), oncolytic adenovirus-derived CG0070, and oncolytic reovirus-derived reolysin (pelareorep) [89].

### 2.5. Cancer Vaccines

Therapeutic cancer vaccines aim to promote tumor regression, establish robust antitumor memory, and avoid adverse events [90]. In principle, cancer vaccines stemmed from the natural phenomenon of antitumor immunity that emerged from natural or chemotherapy-induced immunogenic cell death (ICD). Therapeutic cancer vaccines can be used to treat advanced or relapsed tumors that are refractory to conventional therapies [91]. During ICDs, tumor antigens are released, captured, and cross-presented by APCs, leading to their maturation and migration to secondary lymphoid organs, where they educate naïve T cells. Upon activation, T cells roll back to the TME and cause the direct destruction of cancer cells [92,93].

Cancer vaccines usually contain specific tumor antigens and are exogenously administered to activate APCs such as DCs, leading to the stimulation of an adaptive immune response against tumors containing this antigen and the resurgence of robust tumor control. Usually, large amounts of qualitative antigens are delivered to the DCs to induce optimal DC activation, culminating in sustained T cell activation, TME infiltration, and response maintenance [90]. Alternatively, it is possible to develop a cancer vaccine from an endogenous source, a method called the in situ (ISV) approach. It involves antigen sourcing from dying or dead cells in the TME [94].

Neoantigens derived from tumor mutations have been recognized as ideal targets of T cell-based immunotherapy and therapeutic cancer vaccines [95,96]. Neoantigen-targeted vaccines mainly include synthetic long peptides, nucleic acids, and cell-based vaccines [97]. Currently, many clinical trials have evaluated their safety and efficacy in patients [98,99]. Examples of some vaccines are discussed in the section on clinical trials.

Sipuleucel-T is an antigen-specific active immunotherapy agent that sensitizes the adaptive immune system [100] by activating the anti-PAP (prostatic acid phosphatase) immune response, leading to the destruction of cancer cells [101]. The U.S. FDA approved Sipuleucel-T after a double-blind, placebo-controlled, multicenter phase 3 trial in which Sipuleucel-T reduced the risk of death among patients with metastatic castration-resistant prostate cancer (mCRPC) [102]. Although Sipuleucel-T remains the only cancer vaccine approved, other cancer vaccines are currently being investigated. For instance, four cancer vaccines have been tested in phase 3 clinical trials of mCRPC patients. These include prostate cancer vaccine GVAX (a GM-CSF gene vaccine), anti-prostate-specific antigen (PSA) vaccine PROSTVAC, personalized peptide vaccination (PPV), and DC-based vaccine PCVAC/PCa [101].

In summary, immunotherapy has provided a powerful tool for cancer therapy, either alone or as a synergistic treatment. Some examples of FDA-approved treatments are listed in Table 1.

## 3. Limitations, Challenges, and Solutions to Current Immunotherapy

Even though there are breakthroughs for these immunotherapies, some restrictions or limitations of cancer immunotherapies remain to be overcome, including the development of resistance, treatment efficacy, high treatment cost, and the evaluation of treatment efficacy [104,105]. For example, the top 10 challenges of cancer immunotherapy [106], as well as limitations and potential solutions, are listed in Table 2. In addition, the challenges and potential solutions for each type of immunotherapy are summarized (Table 3).

TME contributes to tumor cell growth and immune evasion [107], limiting the efficacy of immunotherapy. Metabolic restrictions, such as low glucose and pH, hypoxia, and immunosuppressive metabolites in the TME, have important roles in the suppression of anti-cancer therapy, which are new synergistic targets for immunotherapy [108]. For example, challenges for CAR-T cell therapy in solid tumors include tumor heterogeneity or antigen escape, limited tumor infiltration, immunosuppressive TME, induction of CAR-T cell exhaustion, and severe toxicities [109,110]. Potential resolutions for these challenges for CAR T cells include targeting multiple tumor antigens, engineered to secrete anti-tumor cytokines, ICI, and inhibitors for immunosuppressive cytokines (TGF-β1 and IL-4), increasing T cell expansion or persistence, and enhancing tumor infiltration [111].

## 4. Delivery Systems for Immunotherapy

Given that immunotherapy is an important strategy for cancer treatment with various advantages, such as preventing cancer metastasis and recurrence, the above-mentioned limitations, including inefficient delivery system, low efficacy, tumor penetration, optimization of synergistic treatment, off-target effects, and high toxicity of immunotherapeutic agents, can be resolved by delivery systems [121,122,123,124]. In addition, hypoxia, low nutrients in the TME, and the heterogeneity of tumor cells due to mutation significantly inhibit the function of immune cells [125,126]. The delivery system is also a very useful tool for effectively developing a combined therapeutic strategy. For example, therapeutic NPs can be applied to co-deliver chemo-immunotherapy combinations (e.g., doxorubicin and IL-12) to induce efficient intratumor delivery [127].

In this section, we describe some delivery approaches to overcome these limitations and improve cancer immunotherapy, including NP-based delivery, extracellular vesicles, implantable scaffolds, antigen-mediated delivery, and cell-based delivery.

### 4.1. Nanoparticle-Based Delivery

**Figure 2:**
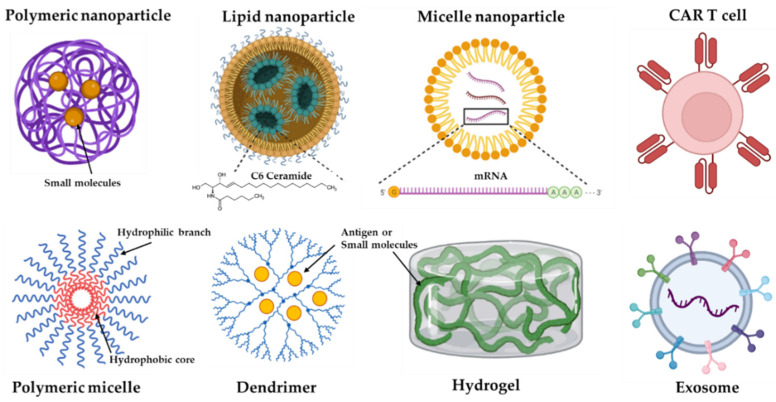
Some representative formats of immunotherapy delivery systems. Nanoparticles (NPs) can be formed by different materials, including iron (e.g., gold), lipid, polymeric, and self-formatting NPs.

NPs can deliver antibodies or their fragments, peptides, proteins, and small molecules and their antagonists, such as IL-2, TGF-β inhibitors, CpG oligodeoxynucleotides, and anti-PD-1 mAbs [128,129]. There are many platforms for NPs, including liposomes, inorganic nanocarriers, dendrimers, polymeric systems, nucleic acid nanotechnology, and exosomes [130]. Some examples are shown in graphic cartoons (Figure 2).

Delivering cancer immunotherapies by NPs can increase anti-tumor efficacy, enhance drug retention, improve drug penetration, and enhance the synergetic effect of treatments [123,131,132]. For example, the self-assembling protein nanocarrier T22-GFP-H6 can selectively deliver cytotoxic agents into CXCR4-expressing tumors in an HNSCC model [133]. Furthermore, the use of NPs overcomes chemotherapeutic resistance by strategies such as inhibition of drug efflux pumps and simultaneous delivery of multiple drugs [134].

#### 4.1.1. Nanovaccines

Cancer vaccines commonly use TAAs and tumor-specific antigens (TSAs) to elicit an anti-tumor immune response to suppress tumor growth [135]. TAAs antigens include overexpressed tumor antigens (e.g., human epidermal growth factor receptor 2, or HER2), cell lineage differentiation antigens (e.g., glycoprotein 100, or gp100), and germline antigens (e.g., melanoma-associated antigen 1, or MAGE-A1) [136]. In contrast, TSA is specifically expressed in tumor cells, but not in normal cells. Vaccine antigens should be delivered to APCs, such as DCs and macrophages, in lymphoid organs [137]. These vaccines can be delivered using different platforms, including cell, virus, peptide, DNA, and mRNA-based vaccines [135].

NPs can also be used as a delivery system for vaccine design, with great interest recently. For example, gold NPs can be used as the core for antigen coating on the surface by stepwise electrostatic interactions between peptide antigens and molecular adjuvant polyanionic toll-like receptor (TLR) agonists. The forming NPs (~40 nm) can be efficiently and primarily internalized by DCs to stimulate the proliferation of antigen-specific T cells and anti-tumor cytokines [138]. A major advantage of using NPs to deliver vaccines is that NPs can be designed according to targeted cells (e.g., DCs) to generate an effective immune response against cancer cells [139]. In addition, it can overcome the limitations of vaccine adjuvants [140].

#### 4.1.2. NP-Loaded Small Molecules

A biodegradable NP consisting of poly-lactic-co-glycolic acid (PLGA) improved cellular uptake and increased the anti-cancer activity of methionine aminopeptidase 2 (MetAp2) inhibitor AD-3281 to melanoma [141]. Loading SB525334, an inhibitor of the transforming growth factor β1 (TGF-β1) receptor, using glutathione-responsive degradable mesoporous silica NPs in TME, induced anti-tumor activity of neutrophils and increased the therapeutic effect of combined irreversible electroporation (IRE) and αPD1 therapy. This resulted from the infiltration of cytotoxic CD8 T cells, depletion of Tregs, and maturation of DCs [142].

An aerosolized star NP can be designed to deliver small interfering RNA (siRNA) aerosol to treat lung cancer in mice [143]. The miktoarm star polymer NPs comprise two main components, poly-dimethylaminoethyl methacrylate (PDMAEMA) and poly [oligo (ethylene glycol) methyl ether methacrylate] (POEGMA), connected via a cystamine-based cross-linker in the core [144]. Self-assembled NPs have also been developed to competently deliver siRNAs intravenously to treat cancers [145]. Intratumoral delivery of lipid nanoparticles (LNPs) encapsulated with IL-12 and IL-27 mRNAs increased infiltration of immune effector cells, including IFN-γ and TNF-α producing NK and CD8 T cells [146]. NPs can maintain the stability of siRNAs or mRNAs and mediate their targeted delivery [147].

From our findings, we also showed that nanoliposome-loaded C6-ceremide (LipC6) can increase tumor cell apoptosis and show a synergistic effect with the adoptive transfer of tumor antigen-specific (TAS) CD8 T cells with subsequent immunization [148]. In addition, this study also showed that LipC6 can not only be used as a delivery system but can also significantly prevent the M2 polarization of TAMs in HCC to induce TSA immune activation [148].

Using synthetic protein nanoparticles (SPNPs)-mediated delivery of CXCR4 antagonist AMD3100 inhibited glioblastoma proliferation by suppressing CXCL2/CXCR4 pathway and reduced the infiltration of CXCR4^+^ monocytic myeloid-derived suppressor cells (M-MDSCs) [149]. Treatment with R848 (an agonist of TLR7 and TLR8)-loaded β-cyclodextrin NPs (CDNP-R848) can drive M1 polarization of tumor-associated macrophages (TAMs) in the TME in multiple mouse tumor models (e.g., tumors caused by mouse colon adenocarcinoma cell line MC-38 and melanoma cell line B16-F10) [150]. In addition, PEG-PLGA NPs can reduce the toxicity of α-PD-L1 F (ab) while maintaining its anti-mouse colon cancer cells MC38 [151].

Natural products, such as polyphenols, show anti-cancer activity in vivo, which can also be delivered by NPs to treat cancer. For example, quercetin shows anti-human breast cancer activity [152]. Treatment with quercetin-conjugated magnetite nanoparticles (QMNPs) inhibited tumor growth and increased the efficacy of lateral radiotherapy treatment in N-methyl-N-nitrosourea-induced breast cancer in female white albino rats [153]. NP-mediated delivery of small molecules can also overcome the dissolving issue, reduce the off-target effect, and improve immunotherapeutic efficacy [148,153,154]. Overall, NPs are useful drug delivery systems for cancer immunotherapy or chemotherapy (Table 4).

### 4.2. Extracellular Vesicles

Extracellular vesicles (EVs) are lipid membrane-enclosed vesicles with nanometer sizes, which are secreted by most living cells, and contain different proteins, lipids, and nucleic acid species of the source cells [155]. These EVs are mediators for the interaction of cells in the TME, regulating anti-tumor immune responses [156]. Given their delivery function, EVs have been explored as carriers of bioactive components of cancer immunotherapy. For example, EVs from fibroblast-like mesenchymal cells can be engineered to deliver siRNAs or short hairpin RNAs (shRNAs) that target oncogenic Kras to enhance anti-pancreatic cancer ability and increase mouse overall survival rates [157].

EVs can be classified into three subtypes, exosomes (30–150 nm), macrovesicles (0.1–1 μm), and apoptotic bodies (1–5 μm) based on their biogenesis mechanism [158]. For example, fibroblast activation protein-α (FAP) gene-engineered tumor cell-derived exosome-like vesicle vaccines (eNVs-FAP) can activate the maturation of DCs, elicit specific cytotoxic T cell infiltration and activation, and promote tumor ferroptosis and depletion of FAP-positive cancer-associated fibroblasts [159]. EVs have many multiple advantages as a delivery platform, including their ability to overcome natural barriers, intrinsic cell targeting properties, and circulation stability [160].

### 4.3. Implantable and Injectable Scaffolds

Conventionally, small drug molecules are dissolved in hydrogel for delivery, which causes drug retention with poor intratumoral delivery. One study applied a nanocomposite hydrogel (~6 nm) to deliver oxaliplatin (OXA) to treat a breast cancer cell line 4T1-induced tumor model. The results showed that this nanocomposite hydrogel significantly decreased tumor growth and metastasis by enhancing the retention and penetration of anti-cancer drugs in the TME, which also showed a synergetic effect with αPD-1 antibody [161]. Another study developed an injectable, polymerized phenylboronic acid-based immunogel for the delivery of mannan, a natural polysaccharide with the function of adjuvanticity and tumor antigen [162]. This immunogel improved anti-cancer activity against a breast cancer cell line 4 T1 cells in a mouse tumor model. Loading NPs in an injectable hydrogel formulation can yield sustained immune stimulation to inhibit cancer cell growth compared to an immediate regular I.V. or I.P. injection [163]. The inverse opal (IOPAL) 3D hydrogels have been engineered with poly(ethylene) glycol (PEG) covalently combined with heparin to resemble the lymph node microenvironment and maintain the phenotype of adoptively transferred T cells [164]. Hydrogel-mediated in situ delivery can provide many advantages, including easy use, increased local treatment agents, and prolonged treatment retention time to prevent the refraction of tumors [165].

### 4.4. Antigen-Mediated Delivery

The self-assembled polysaccharide nanogels of cholesteryl group-modified pullulan (CHP) can be used as antigen delivery systems for cancer immunotherapy by regulating tumor-associated macrophages (TAMs) [137]. New York esophageal squamous cell carcinoma 1 (NY-ESO-1), a cancer-testis antigen, is expressed by many cancers [166]. CHP has been applied to deliver the cancer antigen NY-ESO-1 for cancer vaccines. Patients with advanced or metastatic esophageal cancer were vaccinated with 100 μg or 200 μg of CHP-NY-ESO-1 and showed no adverse events or immunogenicity. The survival of cancer patients increased with a high dose of CHP-NY-ESO-1 treatment compared to low dose administration [167]. This strategy can provide targeted delivery and enhance the immune response [168].

### 4.5. Cell-Based Delivery

T cell transfer therapy, or adoptive cell transfer (ACT) therapy, is a major type of cell-based therapy, including tumor-infiltrating lymphocytes (TIL) therapy and CAR-T cell therapy [169,170]. CAR-T cell therapy is an effective and powerful immunotherapy for combating blood cancers and refractory cancers [171,172]. Unfortunately, it is also very expensive to manufacture CAR T cells [173]. State-of-the-art technology shows that delivery vectors, including lentiviruses, adenovirus-associated vectors, and nanocarriers or NPs, are commonly used for in vivo CAR (encoding nucleic acids) delivery to T cells. Receptor targeting of delivery vectors can reduce off-target cell delivery and potential toxicities [174]. In the above-mentioned CAR-T cell therapy, several products have been approved by the U.S. FDA, including Kymriah for adult patients with relapsed or refractory follicular lymphoma after two or more lines of therapy, Yescarta for adult patients with large B-cell lymphoma that is refractory to first-line chemoimmunotherapy or that relapses within 12 months of first-line chemoimmunotherapy, Tecartus for adult patients with relapsed or refractory mantle cell lymphoma (MCL), Breyanzi for adult patients with relapsed or refractory large B-cell lymphoma after two or more lines of systemic therapy, and Abecma and Carvykti for adult patients with relapsed or refractory multiple myeloma after four or more prior lines of therapy including an immunomodulatory agent, a proteasome inhibitor, and an anti-CD38 mAb (https://www.fda.gov/vaccines-blood-biologics/cellular-gene-therapy-products, accessed on 10 June 2022).

In addition to the above-mentioned delivery systems, drugs themselves can form nanoscale medicines without carriers that have been designed for cancer treatment. Cargo-free nanomedicines can be classified into drug nanocrystals, prodrug self-assembled NPs, drug–drug conjugate NPS, and antibody–drug conjugates (ADCs) [175]. For example, pH-responsive prodrug (PEG–CH=N–Doxorubicin (DOX) has been assembled with the drug SN38 (7-ethyl-10-hydroxycamptothecin) to increase drug accumulation in tumors to kill both non-cancer stem cells and cancer stem cells [176].

## 5. Challenges of Cancer Immunotherapy Delivery Systems

There are some unmet challenges in the delivery of some new therapeutics, such as siRNA-mediated therapeutics due to their low stability and cell uptake. NPs have been widely studied as a delivery system for siRNAs to overcome these unmet challenges [177].

However, CAR-T cell therapy is limited in the treatment of tumors in the central nervous system due to an anatomical barrier that inhibits intratumoral delivery [178]. The safety and efficacy of CAR-T cell therapy and its generation and administration should also be considered [179]. To ensure the therapeutic efficacy of CAR-T cell therapies, its administration may be more appropriate currently in designed centers with high-quality processes and practices [180]. The costs and challenges of CAR-T cell manufacturing require new strategies. One strategy shows that an implantable multifunctional alginate scaffold for T cell engineering and release (MASTER) can decrease the manufacturing time of CAR-T cells in mice. MASTER provides an interface that mediates infection and gene transfer of CD19-encoding retroviral particles to human peripheral blood mononuclear cells with functional CAR-T cell release after implantation [181].

DNA or mRNA vaccines for cancer therapy have several challenges and drawbacks, including insufficient immunogenicity, purification, suboptimal immune system activation, and manufacturing time [182,183].

Oral administration is a widely used method for drug delivery that is limited to cancer therapy. The advanced technique of using nanocarriers, such as liposomes, dendrimers, and solid lipid nanoparticles (SLNs), makes it possible to treat cancer through oral delivery [184].

## 6. Frontiers and Prospects

Intratumoral delivery of many forms of immunotherapy is a promising strategy for improving the efficacy of immunotherapy and minimizing off-target toxicities. For example, oncolytic and non-oncolytic viruses, CAR-T cells, DCs, neoadjuvant immunotherapy, immunostimulatory cytokines, mAbs, and pathogen-associated molecular patterns (PAMPs) can be injected into the tumor to elicit an antitumor immune response [185,186]. This strategy requires relatively low doses of therapeutic agents, reduces off-target side effects, and induces superior T cell priming to kill cancer cells [187]. In addition, intratumoral injection of immunostimulatory agents has shown synergistic effects with other immunotherapies, including ICIs [188]. For example, intratumoral injection of L-pampo, a TLR2/3 agonist, induced a potent T helper cell-mediated immune response and immunogenic tumor cell death, which increased the efficacy of αPD-1 and αCTLA-4 therapies [189]. Currently, many clinical trials are ongoing to evaluate the therapeutic effect of this treatment option and its synergistic effects.

The use of plant virus nanoparticles (PVNPs) for in situ vaccine immunotherapy against cancers is increasing, which has shown considerable effects in preclinical studies. PVNPs, such as cowpea mosaic virus (CPMV) nanoparticles, can be used as adjuvants for cancer vaccines to stimulate an immune response by triggering pattern recognition receptors (PRRs) [163]. PVNPs can also be used as a synergetic strategy to improve local and systemic anti-tumor immunity [190]. In addition, liner DNA amplicons can elicit antigen-specific immunity in animals to show a synergistic effect on ICIs (anti-CTLA-4 or anti-PD-1 antibodies) [191]. Strategies such as the treatment of natural products (e.g., saponins and flavonoids) [192], can be also applied to enhance the efficiency of cancer immunotherapy. For example, the administration of resveratrol induced ovarian carcinoma cell apoptosis and enhanced the infiltration of dendritic cell populations and cytotoxic T cells compared to vehicle treatment [193].

Antibody-mediated targeting delivery can improve the efficacy of NPs. For example, pegylated poly (lactic-co-glycolic acid) (PLGA) NPs encapsulating a combined heparanase T cell epitope alone or in combination with TLR3 and TLR7 ligands can be combined with an anti-DEC-205 (CD205) antibody to target DCs [194]. A study in our lab also showed that LipC6, in combination with an anti-CTLA4 antibody, significantly inhibited HCC growth by increasing the infiltration of CD8 T cells [195].

Furthermore, the approval of CAR-T cells targeting two chimeric antigen receptors by the U.S. FDA for the treatment of hematologic malignancies will improve the application of CAR-T cells in immunotherapy to reduce the limitations caused by antigen escape and off-target side effects [196]. The new generation of CAR-T cells (third and fourth generations) have multiple costimulatory domains and signaling domains, which are engineered into secrete cytokines (e.g., IL-7) and chemokines (e.g., CCL19) [197,198].

Finally, combined cancer therapies can improve anti-tumor immunity and suppress tumor growth. For example, the co-delivery of IL-15 and anti-β-catenin siRNAs with NPs can significantly improve anti-tumor immunity to inhibit tumor growth. This treatment can also prime the effect of the dendric cell vaccine for cancer therapy [199].

## 7. Clinical Trials

Many therapeutics are under clinical evaluation. In this section, we summarize some representative examples of different immunotherapies for cancers in clinical trials (https://www.clinicaltrials.gov/, accessed on 10 June 2022). A summary is listed in Table 5. More extensive studies are needed to translate preclinical research successes into clinical trials and to test their efficacy in patients.

## 8. Conclusions

Currently, there are some breakthroughs in immunotherapeutic treatments, such as the blockade of immune checkpoints and CAR-T cell therapy. Although some barriers hint at their application and efficacy in some solid tumors, many strategies have been shown to promise their application in the clinic. Immunotherapy delivery systems for drugs and biomaterials, such as NPs and implantable scaffolds, have been investigated in the past decade. With advances in these immunotherapeutic strategies and delivery systems, we are close to the next step in cancer treatment. Clinical trials have been investigated, and the promising results will improve therapeutic options for malignant cancers. Improvement of delivery systems and appropriate combination therapies will improve the efficacy of immunotherapy in cancer treatment in the future.

## Figures and Tables

**Table 1:** Some examples of FDA-approved immunotherapies.

S/N	Therapy	Type	Target	Indication	References
1	Ipilimumab	ICI	CTLA-4 blockade	Malignant melanoma	[30]
2	Cemiplimab	ICI	PD-1 blockade	Advanced NSCLC, metastatic CSCC	[39,40]
3	Nivolumab	ICI	PD-1 blockade	Advanced melanoma, metastatic colorectal cancer, NSCLC, renal cell cancer, Hodgkin’s lymphoma, squamous head and neck cancer, urothelial carcinoma, HCC	[36,37]
4	Pembrolizumab	ICI	PD-1 blockade	NSCLC, advanced melanoma, colorectal cancer, gastric and gastroesophageal cancer, classic Hodgkin’s lymphoma, metastatic HNSCC	[41,42,43,44,103]
5	Atezolizumab	ICI	PD-L1 blockade	Triple-negative breast cancer	[45]
6	Durvalumab	ICI	PD-L1 blockade	Urothelial cancer, ES-SCLC	[46,47]
7	Avelumab	ICI	PD-L1 blockade, ADCC	Merkell cell carcinoma, urothelial carcinoma	[48]
8	IFN-α	Cytokine therapy	Multiple mechanisms	mRCC, AIDs-related Kaposi’s sarcoma, follicular lymphoma, chronic myelogenous leukemia, cervical intraperitoneal neoplasms, and advanced melanoma	[26]
9	IL-2	Cytokine therapy	AICD	mRCC	[28,29]
10	Kymriah	ACT	Anti-CD19	B-ALL	[68,69]
11	Yescarta	ACT	Anti-CD19	DLBCL	[68,69]
12	Brexucabtagene autoleucel	ACT	Anti-CD19	R/r mantle cell lymphoma	[39]
13	Tisagenlecleucel	ACT	Anti-CD19	DLBCL, B-ALL, and PMBCL	[40,41]
14	Axicabtagene Ciloleucel	ACT	Anti-CD19	DLBCL, B-ALL, and PMBCL	[40,41]
15	Rigvir	OV	Tumor lysis	Melanoma	[79]
16	Oncorine (H101)	OV	Tumor lysis	Nasopharyngeal carcinoma	[80]
17	Talimogene laherparepvec (T-vec)	OV	Tumor lysis	Melanoma patients	[86]
18	Sipuleucel-T	Cancer vaccine	Activate antitumor immunity	mCRPC	[102]

Abbreviations: ACT: adoptive cell transfer; ADCC: Ab-dependent cell cytotoxicity; B-ALL: B cell acute lymphoblastic leukemia; CSCC: cutaneous squamous cell carcinoma; DLBCL: diffuse large B-cell lymphoma; ES-SCLC: extensive-stage small cell lung cancer; HNSCC: metastatic head and neck squamous cell carcinoma; HCC: hepatocellular carcinoma; ICI: immune checkpoint inhibitor; mCRPC: metastatic castration-resistant prostate cancer; mRCC: metastatic renal cell carcinoma; NSCLC: non-small cell lung cancer; OV: oncolytic virus; PMBCL: primary mediastinal B-cell lymphoma.

**Table 2:** The limitations, challenges, and solutions of cancer immunotherapy.

Cancer Immunotherapy		References
Limitations	Unpredictable efficacy Clinically significant biomarkers Tumor heterogeneity Acquired treatment resistance Clinical trial design Delivery system Cost of cancer immunotherapy	[9,104,105,112]
Challenges	Developing preclinical models for drug discovery and evaluation Determining the specific drivers of cancer immunity Understanding organ-specific tumor immune contexture Understanding the underlying mechanism of primary immune escape compared to secondary immune escape Illustrating the benefits of endogenous versus synthetic immunity Effectively and efficiently evaluating combinational immunotherapies in early-phase clinical studies Fully characterizing the impact of steroids and immune suppression on immunotherapy and autoimmune toxicities Maximizing personalized approaches through composite biomarkers Improving regulatory endpoints for immunotherapy Optimizing long-term survival with multi-agent combination regimens	[106]
Solutions	Identification of cancer genetic mutations, biomarkers, tumor antigens, and development pathways Combinational treatments and multiple antigen-targeted treatments Conduct pre-clinical and clinical trials Precision treatment by characterizing tumor heterogeneity Identifying and targeting resistant tumor cells Pre-screening by predictive markers and using immunoprotective treatments to decrease costs Developing an effective delivery system for cancer immunotherapy	[104,113]

**Table 3:** Challenges and potential solutions for each type of immunotherapy.

S/N	Types of Immunotherapies	Challenges	Potential Solutions	References
1	ICI	Lack of biomarkers that can predict therapeutic response Inadequate robust clinical strategies for the development of combination therapies Immune-related adverse events (irAEs) Inefficient delivery system due to impenetrable dense stroma	Development of predictive biomarkers Improvement of the clinical approach to the development of combination therapies The use of ICIs in combination with drugs that prevent irAEs The use of ICIs in combination with stroma-degrading therapies	[26,114]
2	Cytokine therapy	High toxicity Low efficacy	Enhancement of local administration strategies Optimization of combination strategies Solution to adverse interactions with TME	[115]
3	ACT	Modest anti-tumor activities Antigen escape High toxicity Restricted trafficking Host–TME interaction with CAR T cells Limited tumor infiltration	Improvement of engineering strategies for CAR T cells development Alteration of CAR structure by decreasing the affinity of antigen-binding domains to lower toxicity The use of humanized antibody fragments rather than murine-derived	[116]
4	OV	Antiviral immune response Off-target infection Adverse effects Ineffective delivery system Lack of specific predictive biomarkers	The use of ECM modulators Capsid modifications The use of cellular carriers Combination with anti-angiogenic agents Better selection of reliable biomarkers	[117]
5	Cancer vaccine	Instability Inefficient delivery system Innate immunogenicity	Structural modification such as codon expansion or optimization in the case of mRNA vaccine Improvement of formulation methods	[118,119,120]

Abbreviations: ACT: adoptive cell transfer; CAR: chimeric antigen receptor; OV: oncolytic virus; TME: tumor microenvironment.

**Table 4:** Nanoparticles for drug delivery in cancer treatment.

Cancers	Nanoparticles	Drugs	Effect	References
Hepatocellular carcinoma	Polymeric	Bortezomib	Sustain release of Bortezomib for 30 days.	[131]
	Lipid	C6-ceremide	Nanoliposome-loaded C6-ceremide (LipC6) increased activation of TAS CD8 T cells and induced M1 polarization of tumor-associated macrophages (TAMs).	[148]
Melanoma	Polymeric	AD-3281	Improve cellular uptake of methionine aminopeptidase 2 inhibitor AD-3281 and its anti-cancer activity.	[141]
Pancreatic cancer	Mesoporous silica	SB525334	Loading SB525334, an inhibitor of transforming growth factor β1 (TGF-β1) receptor, using glutathione-responsive degradable mesoporous silica nanoparticles in tumor microenvironment induced anti-tumor activity of neutrophils and increased the therapeutic effects of combined irreversible electroporation (IRE) and αPD1 therapy.	[142]
Lung cancer	Polymeric	siRNAs	Inhibit expression of βIII-tubulin and Polo-Like Kinase 1 (PLK1).	[143]
Melanoma	Lipid	mRNAs	Intratumoral delivery of lipid nanoparticles (LNPs) encapsulated with IL-12 and IL-27 mRNAs increased infiltration of immune effector cells, including IFN-γ and TNF-α producing NK and CD8 T cells.	[146]
Colon cancer	Polymeric	α-PD-L1	The α-PD-L1 F(ab)-PEG-PLGA nanoparticle (α-PD-L1 NP) is a non-toxic NP that can extend α-PD-L1 antibody circulation time while keeping its anti-cancer activity against mouse colon cancer model (MC38).	[151]
Glioblastoma	Synthetic protein	AMD3100	Using synthetic protein nanoparticles (SPNPs)-mediated delivery of CXCR4 antagonist AMD3100 inhibited the CXCL2/CXCR4 pathway in glioblastoma proliferation and reduced the infiltration of CXCR4^+^ monocytic myeloid-derived suppressor cells (M-MDSCs).	[149]
Multiple tumor models	Cyclodextrin	R848	Treatment with R848, an agonist of the toll-like receptors TLR7 and TLR8, mediate M1 polarization of TAMs.	[150]
Breast cancer	Magnetite	Quercetin	Treatment with quercetin-conjugated magnetite nanoparticles (QMNPs) inhibited tumor growth and increased the efficacy of lateral radiotherapy treatment in N-methyl-N-nitrosourea-induced breast cancer in female white albino rats.	[153]

**Table 5:** Strategies for immunotherapy and treatment delivery.

Clinical Trials	Phase	Treatment	Therapy	Results	References
NCT01491893	1	Intratumoral delivery of the recombinant nonpathogenic polio-rhinovirus chimera	Viral	The survival rate among patients with recurrent grade IV malignant glioma who received PVSRIPO immunotherapy was higher at 24 and 36 months than the rate among historical controls.	[200]
NCT01052142	1	Lipovaxin-MM, a novel dendritic cell-targeted liposomal vaccine	Vaccine	It was well tolerated and did not induce clinically significant toxicity. Partial response and stable disease were observed in one and two patients, respectively.	[201]
NCT03874897	1	Claudin18.2 (CLDN18.2)-redirected CAR T cells	CAR-T	Treatment of Claudin18.2 (CLDN18.2)-targeted CAR T cells showed promising efficacy with an acceptable safety profile in pretreated patients with CLDN18.2-positive digestive system cancers.	[202]
NCT03182816	1	Infusions of piggyBac transposon system-generated EGFR-CAR-T cells	ACT	Non-viral piggyBac transposon system-engineered EGFR-CAR-T cell therapy is feasible and safe in the treatment of EGFR-positive advanced relapsed/refractory NSCLC patients.	[203]
NCT 02348216	2	Axicabtagene ciloleucel (axi-cel), an autologous anti-CD19 CAR T cell therapy	ACT	Patients with refractory large B-cell lymphoma from a multicenter study showed a high-level durable response to axicel therapy.	[72]
NCT01174121	2	Immunotherapy using tumor-infiltrating lymphocytes (TILs) for patients with metastatic breast cancer	ACT	Adoptive transfer of TILs showed objective complete and partial responses in this pilot study.	[204]
NCT02858895	2	IL-4R-targeted immunotoxin (MDNA55)	Cytokine	Treatment of MDNA55, a fusion protein comprising a genetically engineered IL-4 linked to a modified version of the *Pseudomonas aeruginosa* exotoxin A (PE) that binds to the IL-4 receptor (IL-4R) in cancer cells and non-malignant immunosuppressive cells, was associated with progression-free (PFS) and overall survival (OS) in recurrent glioblastoma (rGBM) detected by the modified radiographic response assessment in neuro-oncology (mRANO).	[205]
NCT02843204	2	Pembrolizumab plus NK cell therapy	ICI & cell	Pembrolizumab (αPD-1 antibody) plus NK cell therapy increased overall survival and progression-free survival times in patients with advanced NSCLC and previous PD-L1 treatment.	[206]
NCT01967823	2	Adoptive transfer of autologous T cells transduced with a T cell receptor (TCR)	ACT	T cell receptor immunotherapy targeting NY-ESO-1 for patients with metastatic melanoma and synovial cell sarcoma.	[207,208]
NCT03196830	2	Anti-CD30 CAR-T treatment combined with a PD-1 inhibitor	ACT	The combined treatment with αPD-1 antibody and CD30 CAR-T therapy showed a synergistic effect in relapsed/refractory CD30^+^ lymphoma patients, without causing severe toxicities.	[209]
NCT01245673	2	Autologous stem cell transplant (ASCT)	Cell	A specific T cell response was induced after infusion of autologous T cells with a MAGE-A3 multipeptide vaccine (compound GL-0817) combined with Poly-ICLC (Hiltonol) and GM-CSF.	[210]
NCT01159288	2	Dendritic cell-derived exosomes (Dex)	Neoantigen	Using IFN-γ-Dex loaded with MHC class I- and class II-restricted cancer antigens showed the capability to increase the anti-tumor immunity of NK cells in patients with advanced NSCLC.	[211]
NCT02425891	3	Atezolizumab (αPD-L1 antibody) plus nab-paclitaxel	ICI and chemotherapy	Atezolizumab plus nab-paclitaxel prolonged PFS among patients with metastatic triple-negative breast cancer in both the intention-to-treat population and the PD-L1-positive subgroup.	[212]

## Data Availability

All data supporting reported results can be found in this manuscript.
